# The Pleasantries of the Mob

**Published:** 1892-09-17

**Authors:** 


### PASSING TOPICS.

### THE PLEASANTRIES OF THE MOB.

No Englishman possessing an atom of chivalry, or even of that nineteenth century product which does duty for the more sterling article, can have read of the disgraceful scene at Hendon over M. Carpazza's parachute fiasco without a feeling of shame. We are so often told by apologist writers that our crowds are essentially good humoured, that we want to know why upon occasions, with very trifling provocation or with none, an English mob displays a coarse, unreasoning brutality such as no other under the sun would descend to? In the present case, which we only allude to as a current illustration, there was nothing to account for, much less to justify, the attitude adopted towards the unoffending Frenchman, whose good faith has not been impugned, and who was at least as much a victim to circumstances as any one of those whose stake in the venture was limited to the shilling they had paid for admission to the ground. It was no error or neglect of his which brought about the accident to his apparatus. No one suggested it was, yet his failure to carry out the programme under conditions not of his making, and which rendered progress impossible, was deemed a sufficient reason for offering violence to the unhappy exhibitor, who was only saved from grave consequences by the prompt action of the authorities. No wonder M. Carpazza had little desire to renew the acquaintance of the howling horde of savages who, so far as his experience went, were representative of the great English nation, and that he went back to Paris at the earliest opportunity, carrying with him notions the reverse of flattering concerning the civilisation of these islands.

It is, perhaps, no very remarkable matter that English society should be wronged in this particular instance. A foreigner would hardly discriminate between one and another gathering of English people. Otherwise, M. Carpazza might have known that most of the persons who paid a shilling to witness his performance were attracted chiefly by the chance of seeing him break his neck, and if this could have been reduced to a certainty, they would gladly have paid two shillings. But although we may not be concerned to offer elaborate explanations to M. Carpazza, we ought at least to be solicitous of the reputation of Englishmen generally in respect of their treatment whether of foreigners or of one another. In the metropolis and other large cities, and even in small provincial towns, there is always a certain section of the population which makes itself unpleasantly conspicuous, and to which the field is so commonly surrendered, that it has come to regard possession as a right, and to work out its own sweet will in its own way, without fear of hindrance from the more respectable portions of society.

We think the weakly amiable propensity to be blind and deaf to what is unpleasant, is carried to an extreme, with the result that the dregs of the population are obtrusively in evidence everywhere. Even when well-behaved people congregate in force they are equally pusillanimous. Filthy language and rude behaviour are endured without a protest for fear that worse may ensue, or if found unendurable, the victims are content to move a few paces onward in silence. England would be a very much pleasanter place if the decent people who form the overwhelming majority would pluck up courage to stamp out the ruffianism which now exercises itself with impunity upon inoffensive persons, native and foreign.

Agricola.

